# Assessment of macular and peripapillary choroidal thickness in non-arteritic anterior ischemic optic neuropathy: A meta-analysis

**DOI:** 10.1097/MD.0000000000032916

**Published:** 2023-02-22

**Authors:** Xuejiao Li, Haoliang Chen, Yalong Dang

**Affiliations:** a Department of Ophthalmology, Sanmenxia Central Hospital, Sanmenxia, Henan, China; b Department of Neuro-ophthalmology, Sanmenxia Eye Hospital, Sanmenxia, Henan, China; c Department of Ophthalmology, Henan University of Science and Technology School of Medicine, Luoyang, Henan, China.

**Keywords:** choroidal thickness, meta-analysis, non-arteritic anterior ischemic optic neuropathy, optical coherence tomography

## Abstract

**Background:**

Non-arteritic anterior ischemic optic neuropathy (NAION) is the most common optic neuropathy in adults aged ≥ 50 years. Transient non-perfusion or hypoperfusion of the optic nerve head circulation is believed to be the underlying cause of NAION. It has been suggested that peripapillary choroidal thickness (PCT) is altered after ischemic disorders of the optic nerve head, but the results have not always been consistent. To address this issue and provide evidence for the pathogenesis of NAION, we performed a meta-analysis to systematically evaluate macular choroidal thickness (MCT) and PCT in patients with NAION.

**Methods:**

A comprehensive literature search of PubMed, Embase, Cochrane Library, and Web of Science databases was performed until August 31, 2022. The main inclusion criterion was a case-control study in which MCT and PCT were measured using optical coherence tomography in patients with NAION. Mean difference (MD) and 95% confidence interval were calculated for continuous estimates. The Review Manager (V5.40) was used for the analysis.

**Results:**

Nine studies comprising 663 eyes (283 NAION eyes and 380 healthy control eyes) were included (Newcastle-Ottawa Scale score ≥ 5). MCT and PCT were higher in eyes with chronic NAION (MD = 19.16, *P *= .04; MD = 35.36, *P *< .00001) and NAION fellow eyes (MD = 30.35, *P *= .0006; MD = 29.86, *P *= .04) than in healthy controls. No difference was noted in the MCT between eyes with acute NAION and healthy controls (MD = 2.99, *P *= .87).

**Conclusion:**

Increased MCT and PCT may be important anatomical and physiological features of the eyes in patients with NAION.

## 1. Introduction

Non-arteritic anterior ischemic optic neuropathy (NAION) is the most common optic neuropathy in adults aged ≥ 50 years, with an annual incidence of 2.3 to 82 per 100,000 individuals.^[1–3]^ Patients typically present with acute, painless, unilateral loss of vision associated with a variable visual field defect, relative afferent pupillary defect, swollen and hyperemic optic disc, and 1 or more flame-shaped peripapillary retinal hemorrhages.^[3]^ The risk factors for NAION include male sex, hypertension, hyperlipidemia, diabetes mellitus, coronary heart disease, sleep apnea, medication history of cardiovascular drugs, and factor V Leiden heterozygosity.^[4]^ In addition, transient non-perfusion or hypoperfusion of the optic nerve head (ONH) circulation is believed to be the underlying cause of NAION.^[5]^

The choroid of the eye is primarily a vascular structure that supplies oxygen and nutrients to the outer retina.^[6]^ Choroidal changes may be involved in retinal and optic nerve diseases such as NAION, because fine centripetal branches supply the anterior part of the optic nerve from the peripapillary choroid and centripetal branches of the short posterior ciliary artery, either directly or by the arterial circle of Zinn and Haller.^[5]^ Therefore, it has been suggested that peripapillary choroidal thickness (PCT) is altered after ischemic disorders of the ONH.^[5]^ However, there is no equivalent imaging device to study retinal vessels in clinical practice. Choroidal imaging is limited to fluorescein angiography, indocyanine green angiography, laser Doppler flowmetry, and high-resolution ultrasonography, with marked restrictions in resolution and the ability to differentiate structures. Since Spaide et al^[7]^ introduced enhanced depth imaging-optical coherence tomography (OCT) based on spectral-domain OCT technology, an increasing number of investigators have studied choroidal thickness in eyes with NAION to reveal the pathogenesis of NAION.^[7–18]^ Some studies have found that macular choroidal thickness (MCT) and PCT significantly decreased in eyes affected by NAION compared with those in healthy control eyes,^[7,10,17]^ whereas other studies have reported controversial results.^[9,11–16,18]^

To address this issue and provide evidence for the pathogenesis of NAION, we performed a meta-analysis to systematically evaluate MCT and PCT in patients with NAION. To the best of our knowledge, this is the first meta-analysis to evaluate MCT and PCT in NAION.

## 2. Materials and Methods

### 2.1. Ethnic statements, search, and identification strategy

This study was performed in accordance with the preferred reporting items for systematic reviews and meta-analyses statement and was registered in PROSPERO (registration number CRD42022369043). ethical approval was not required.

Two independent investigators (L.X. and C.H.) comprehensively searched electronic databases, including PubMed, Embase, Cochrane Library, and Web of Science, to identify relevant literature. The final search was conducted on August 31, 2022. We created our search strategy following the population-intervention-control outcomes framework: (P) our population consisted of patients with NAION, (I) who had undergone an OCT examination, (C) no settings, (O) outcomes were choroidal thicknesses; the search terms were applied as follows: (“Optic Neuropathy, Ischemic” or “Optic Ischemic Neuropathy” or “Ischemic Neuropathy, Optic” or “Neuropathy, Optic Ischemic” or “Optic Ischemic Neuropathies” or “Optic Nerve Ischemia” or “Ischemia, Optic Nerve” or “Nerve Ischemia, Optic” or “Optic Nerve Ischemias” or “Optic Ischaemic Neuropathy” or “Ischaemic Neuropathy, Optic” or “Neuropathy, Optic Ischaemic” or “Optic Ischaemic Neuropathies” or “Ischemic Optic Neuropathy” or “Ischemic Optic Neuropathies” or “Neuropathy, Ischemic Optic” or “Posterior Ischemic Optic Neuropathy” or “Optic Neuropathy, Posterior Ischemic” or “Nonarteritic Anterior Ischemic Optic Neuropathy” or “NAION” or “Anterior Ischemic Optic Neuropathy” or “Optic Neuropathy, Anterior Ischemic”) and (“Tomography, Optical Coherence” or “Coherence Tomography, Optical” or “OCT Tomography” or “Tomography, OCT” or “Optical Coherence Tomography”) and (“choroidal thickness”). The language used was restricted to english. These search terms were used for all article fields and were not limited to their abstracts. No search filters were used in this study. After removing duplicates automatically and manually, the studies were screened by title, abstract, and full text.

### 2.2. Inclusion and exclusion criteria

Studies were included in this meta-analysis if they met the following criteria: they were original articles; they were concerned with comparisons of choroidal thickness between NAION (including acute and chronic stages) patients and controls; and choroidal thickness data were provided as mean ± standard deviation that could be extracted.

The exclusion criteria were as follows: article types were case series, abstracts, posters, animal studies, reviews, comments, editorials, and meta-analyses; the imaging technique was OCT optical coherence tomography angiography (OCTA); duplicate publications from the same study; and low-quality studies (newcastle-ottawa scale [NOS] stars < 5).

### 2.3. Data extraction

Two investigators (L.X. and C.H.) independently extracted the data from the included studies, and disagreements were resolved by a third reviewer or via an open discussion. The following data were collected: first author, country, year of publication, study design, number of eyes, mean age, sex, time of onset of NAION, device, and outcomes.

### 2.4. Quality assessment

The NOS, which provides a score range of 0 to 9 stars, was used for the quality assessment. The NOS assigns a higher score to blinded exposure assessment studies; however, blinding is sometimes impossible because the case-control status can be easily discerned owing to optic disc edema.^[19]^ Therefore, studies scoring 5 or more stars were considered to be of better quality and were included in the final analysis. Any different views on this process were addressed and consulted by a third investigator.

### 2.5. Statistical analysis

Review manager version 5.40 (Cochrane Collaboration, Oxford, UK) was used for data analyses. Mean difference (MD) and 95% confidence interval (CI) were calculated for continuous estimates. Heterogeneity between studies was determined using the chi-square test based on the *Q* and *I^2^* values. Considering *P*  < .05 or an *I^2^* value of > 50%, represents statistical significance, a random-effects model was used; otherwise, a fixed-effects model was used. *I^2^* values of 25%, 50%, and 75% represented mild, moderate, and high heterogeneity, respectively. The results were considered significant if the *P*  < .05. To avoid the possibility of drawing a misleading conclusion, potential publication bias was not tested because the number of studies included was low (<10).^[20]^ A sensitivity analysis was performed using the leave-1-out method.

## 3. Results

### 3.1. Identification of studies

**Figure 1.**
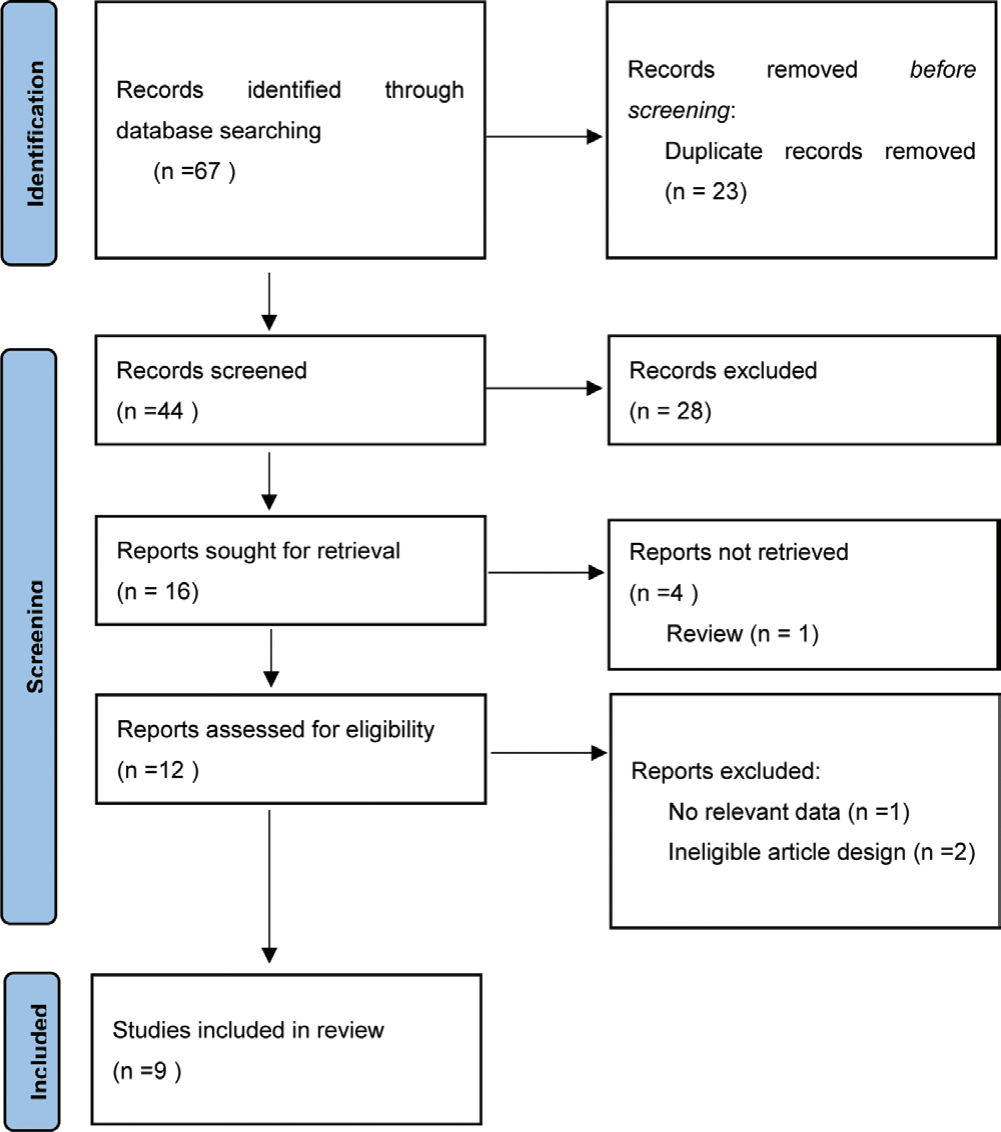
Flow diagram of the literature search and selection process.

A total of 67 potential articles were initially identified from the databases (PubMed,16; Web of Science,16; Embase,35; Cochrane Library,0), of which 23 duplicate articles were excluded. In addition, 28 articles were excluded after reviewing their titles and abstracts. The full text of the remaining 16 articles was screened in detail; 4 articles did not meet the inclusion criteria. The process of verifying the studies was in accordance with the preferred reporting items for systematic reviews and meta-analyses flow diagram (Fig. 1). Finally, 9 studies containing 663 eyes (283 in the NAION group and 380 in the control group) met the inclusion criteria. Therefore, they were included in our meta-analysis.^[7–15]^

### 3.2. Baseline characteristics and quality of included studies

The basic characteristics of the included studies are summarized in Table 1. The studies were published between 2014 and 2021. Among them, 2 studies were performed in Spain, 2 in China, 2 in Iran, 1 in Turkey, 1 in Germany, and 1 in Lisbon. For choroidal thickness measurement, 6 studies used Heidelberg OCT, 1 used Topcon OCT, 1 used Carl Zeiss OCT, and 1 used OptoVue OCT.

**Table 1:** Baseline characteristics of included studies.

	Included study	Country	Study design	Number of eyes			Mean age (yr)			Gender(M/F)			Onset time of NAION	Device	Outcomes
				Group 1	Group2	control	Group 1	Group2	control	Group 1	Group2	control			
1	García-Basterra I et al 2016	Spain	NA	22 (chronic)		42	64.4 ± 11.4		64.0 ± 7.8	14/8		21/21	Group 1 > 3 months	SD-OCT	MCT
2	Gonul S et al 2016	Turkey	case–control study	23 (chronic)	24 (acute)	24	62.17 ± 7.01	61.62 ± 6.99	61.62 ± 6.99	13/10	13/11	13/11	Group 1: 23.86 ± 16.70 months Group 2: 7.45 ± 8.86 days	SD-OCT	MCT
3	Jiang L et al 2016	China	observational case–control study	19 (acute)	25 (chronic)	60	50.63 ± 10.92	51.00 ± 9.37	50.30 ± 1.20	10/9	13/12	30/30	NA	SD-OCT	PCT,MCT
4	Schuster AK et al 2014	Germany	retrospective observational study	20 (acute)		58	66.9 ± 9.8		71.9 ± 11.7	16/4		NA	NA	SD-OCT	MCT
5	Nikkhah H et al 2020	Iran	prospective comparative case-control study	38 (acute)	38 (fellow)	74	62 ± 11	62 ± 11	61 ± 30	20/18	20/18	18/19	Group 1 < 14 days	SD-OCT	PCT,MCT
6	Pérez-Sarriegui A et al 2018	Spain	observational cross-sectional study	29 (chronic)	21 (fellow)	29	67.20 ± 11.25	66.47 ± 11.49	68.03 ± 12.11	16/13	12/9	12/17	Group 1 > 6 months	SS-OCT	PCT,MCT
7	Hou YT et al 2021	Taiwan	observational study	35 (chronic)	29 (fellow)	40	59.97 ± 8.27	NA	59.08 ± 14.92	19/13	NA	19/21	Group 1: 142 days	SD-OCT	PCT
8	Fard MA et al 2015	Iran	prospective, comparative study	30 (chronic)	30 (fellow)	25	56 ± 9.9	56 ± 9.9	57.5 ± 9.8	15/15	15/15	15/10	Group 1: 4.9 ± 0.9 months	SD-OCT	PCT
9	Dias-Santos A et al 2014	Lisbon	cross-sectional study	19 (chronic)		28	65.25 ± 12.76		69.95 ± 10.28	6/12		5/23	NA	SD-OCT	MCT

M/F = Male/ female, MCT = macular choroidal thickness, NA = not available, NAION = non-arteritic anterior ischemic optic neuropathy, PCT = peripapillary choroidal thickness, SD-OCT = spectral-domain optical coherence tomography, SS-OCT = swept-source optical coherence tomography.

The results of quality assessment of the included studies are presented in Table 2. Six studies were graded higher than 6 stars and 3 had 5 stars.

**Table 2:** NOS for assessing study quality.

	Study	Selection	Comparability	Exposure	Total score
1	García-Basterra I et al 2016	3	1	3	7
2	Gonul S et al 2016	2	1	2	5
3	Dias-Santos A et al 2014	2	1	2	5
4	Pérez-Sarriegui A et al 2018	3	1	2	6
5	Jiang L et al 2016	4	1	3	8
6	Hou YT et al 2021	4	1	2	7
7	Schuster AK et al 2014	3	1	1	5
8	Nikkhah H et al 2020	4	1	2	7
9	Fard MA et al 2015	4	1	3	7

NOS = newcastle-ottawa scale.

### 3.3. MCT increased in chronic NAION eyes but not in acute NAION eyes compared with healthy controls

**Figure 2.**
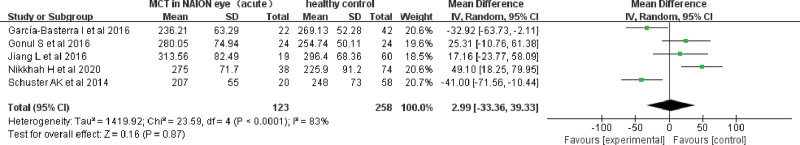
Forest plot showing MCT in acute NAION groups and control groups. MCT = macular choroidal thickness, NAION = non-arteritic anterior ischemic optic neuropathy.

**Figure 3.**
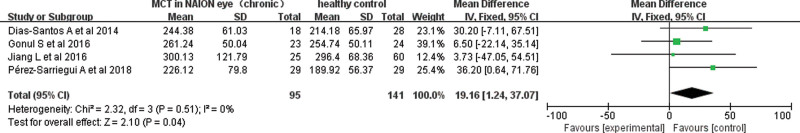
Forest plot showing MCT in chronic NAION groups and control groups. MCT = macular choroidal thickness, NAION = non-arteritic anterior ischemic optic neuropathy.

MCT in acute NAION was studied in 5 of the 9 original studies,^[7–11]^ including 381 eyes (123 in the acute NAION group and 258 in the control group). Four of the 9 original studies,^[8,9,12,15]^ including 236 eyes (95 in the chronic NAION group and 141 in the control group), reported MCT in chronic NAION. Our results showed that the pooled MD for MCT between the acute NAION and control groups was 2.99 (95% confidence interval [CI: −33.36 to 39.33, *P* = .87, Fig. 2), with high heterogeneity (Χ^2^ = 23.59, *P* < .0001, *I^2^* = 83%, Fig. 2), showing that there was no significant difference in MCT between the acute NAION and control groups. The pooled MD for MCT between the chronic NAION and control groups was 19.16 (95% CI: 0.64–71.76, *P* = .04; Fig. 3), showing that MCT was higher in the chronic NAION group than in the control group without heterogeneity among these studies (Χ^2^ = 2.32, *P* = .51, I^2^ = 0%; Fig. 3).

### 3.4. MCT was higher in NAION fellow eyes than in controls

**Figure 4.**
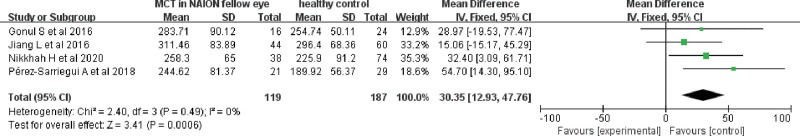
Forest plot showing MCT in NAION fellow eye groups and control groups. MCT = macular choroidal thickness, NAION = non-arteritic anterior ischemic optic neuropathy.

Four of the 9 original studies were included in the analysis of MCT in NAION fellow eyes and healthy controls,^[8,9,11,12]^ including 306 eyes (119 in the NAION group and 187 in the control group). The pooled MD for MCT between the 2 groups was 30.35 (95% CI: 122.93–47.76, *P* = .0006; Fig. 4), with no heterogeneity across studies (Χ^2^ = 2.40, *P *= .49, *I^2^* = 0%; Fig. 4), showing that MCT was higher in NAION fellow eyes than in control eyes.

### 3.5. PCT was higher in chronic NAION than in controls

**Figure 5.**
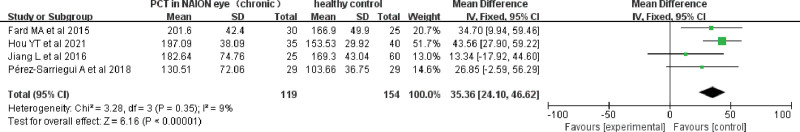
Forest plot showing PCT in chronic NAION groups and control groups. PCT = peripapillary choroidal thickness, NAION = non-arteritic anterior ischemic optic neuropathy.

Four of the 9 original studies were included in the analysis of PCT in chronic NAION,^[9,12–14]^ including 273 eyes (119 in the chronic NAION group and 154 in the control group). Only 2 of the 9 original studies reported PCT levels in acute NAION; therefore, we could not analyze this outcome. The pooled MD for PCT between the chronic NAION and control groups was 35.36 (95% CI: 24.10–46.62, *P* < .00001, Fig. 5), with mild heterogeneity across studies (Χ^2^ = 3.28, *P *= .35, *I^2^* = 9%, Fig. 5), indicating that PCT was higher in the chronic NAION group than in the control group.

### 3.6. PCT was higher in NAION fellow eyes than controls

**Figure 6.**
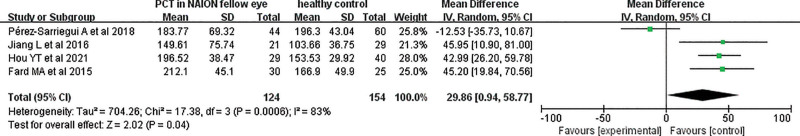
Forest plot showing PCT in NAION fellow eye groups and control groups. NAION = non-arteritic anterior ischemic optic neuropathy, PCT = peripapillary choroidal thickness.

Four of the 9 original studies were included in the analysis of PCT in NAION fellow eyes compared with that in healthy controls,^[9,12–14]^ including 278 eyes (124 in the NAION group and 154 in the control group). The pooled MD for PCT between these 2 groups was 29.86 (95% CI: 0.94–58.77, *P *= .04, Fig. 6), with high heterogeneity across studies (Χ^2^ = 17.38, *P *= .0006, *I^2^* = 83%, Fig. 6), indicating that PCT was higher in the NAION group than in the control group. However, sensitivity analyses were performed in this group, and the heterogeneity decreased remarkably from 83% to 0% (Χ^2^ = 0.03, *P* = .98) when the study by Pérez-Sarriegui et al was excluded.

## 4. Discussion

To the best of our knowledge, this is the first meta-analysis to investigate MCT and PCT in patients with NAION. Our meta-analysis included 9 original articles on MCT and PCT in patients with NAION and healthy controls. Nine eligible studies, including 663 eyes, were analyzed in this meta-analysis and the MCT and PCT data were pooled. Our data showed 2 main results regarding NAION: MCT and PCT were higher in eyes with chronic NAION and NAION fellow eyes than in healthy controls; and no difference was noted in MCT between eyes with acute NAION and healthy controls. However, PCT in acute NAION has not been analyzed because only 2 original studies have reported it.

According to our results, the MCT was higher in the chronic NAION group than in the control group; however, this difference was not noted in the acute NAION group. The MCT was significantly higher in NAION fellow eyes than in healthy controls. In the comparative analysis of MCT in eyes with acute NAION and healthy controls, substantial heterogeneity was observed among the 5 included studies with an *I^2^* value of 83% (*P* < .0001), and the majority of heterogeneity was from the studies by García-Basterra et al^[7]^ and Schuster et al^[10]^ Heterogeneity was significantly decreased when these 2 studies were excluded (*I^2^* = 0%, *P* = .41), and pooled MD increased to 33.64 (95% CI: 13.30–53.98, *P* = .001), indicating that MCT was higher in eyes with acute NAION than in healthy controls. Sensitivity analyses indicated no difference in MCT between eyes with acute NAION and healthy controls, which was not robust. These 2 studies reported lower subfoveal choroidal thickness in the affected and unaffected fellow eyes of patients with NAION. These conflicting results may be attributed to differences in the patient populations. NAION patients with diabetes mellitus were included in studies by García-Basterra et al and Schuster et al A previous study showed that the eyes of patients with diabetes and the presence of diabetic retinopathy were associated with decreased central choroidal thicknesses.^[21]^ Regarding age, patients in the studies by García-Basterra et al and Schuster et al were relatively older than those in other studies (64.4 ± 11.4 and 66.9 ± 9.8, respectively). It had been reported that for each year increase in age, the subfoveal choroidal thickness decreased by 4.1 µm.^[22]^ Among the included studies, there were differences in measurement methods, devices used, and manual segmentation. Most studies used a single horizontal line or multiline scan focused on the fovea to measure MCT; 1 study used an ETDRS grid that could automatically segment the macular area to measure the regional mean choroidal thickness.^[12]^

Similarly, different OCT devices can produce different outcomes.^[23]^ Our results indicated that increased MCT in eyes with chronic NAION and fellow eyes with NAION might be a structural predisposing factor due to ischemia. There is a variable risk of NAION in the fellow eye (15%–19%) over the subsequent 5 years.^[24]^ The pooled result of MCT in eyes with acute NAION was not robust; therefore, more original studies are needed for further validation.

PCT was significantly higher in chronic NAION and NAION fellow eyes than in the healthy control eyes. However, substantial heterogeneity was observed in the comparative analysis of PCT levels in fellow NAION and control eyes. Pérez-Sarriegui et al contributed to this major heterogeneity. The *I^2^* value for this item significantly decreased from 83% to 0% (Χ^2^ = 0.03, *P *= .98) after the removal of this study. Although this study was excluded from the analysis, the MD estimate of this parameter was still significantly different (*P* < .00001), suggesting that the results were robust. Circulatory insufficiency in the ONH is a known cause of NAION, which is associated with dysfunction of the short posterior ciliary arteries. Some studies that expected changes in PCT indicated dysfunction or insufficiency of the ONH. Our results suggest a thicker peripapillary choroid in fellow eyes with chronic NAION and NAION, which is consistent with the findings reported by Jiang et al,^[9]^ Pérez-Sarriegui et al,^[12]^ Hou et al,^[13]^ and Fard et al 2014, who indicated that PCT was higher in both eyes of patients with NAION. Nikkhah et al^[11]^ (2020) compared PCT levels in eyes with acute NAION with healthy controls and reported higher PCT in eyes with acute NAION and uninvolved fellow eyes. We did not measure PCT in patients with acute NAION because of insufficient raw data. Nikkhah et al suggested that a thicker peripapillary choroid in eyes with NAION may be a predisposing structural factor.^[11]^ Fard et al suggested that a thick choroid may have been present prior to the onset of NAION.^[14]^ Pérez-Sarriegui et al^[12]^ reported an automated evaluation method for choroidal thickness in NAION that demonstrated greater measurement accuracy. They suggested that a thicker choroid is not the cause of NAION.

In contrast, Jiang et al found that PCT was higher in the nasal, nasal inferior, and temporal inferior segments of eyes with optic disc edema than in healthy controls, suggesting that abnormal thickening of the peripapillary choroid in some segments can be pathologically attributed to optic disc edema in the early stage of NAION, rather than contributing to the development of NAION.^[9]^ They inferred that these alterations in PCT levels were secondary effects rather than the cause of NAION. Meanwhile, some studies suggested that choroidal thickening, as an additional factor, contributes to the structural crowding in NAION, which was called “compartment syndrome.”^[25]^ Our results support the view that increased PCT might be a predisposing factor for NAION onset rather than a subsequent result. Considering PCT in fellow eyes with NAION, we believe that increased PCT is a morphological feature associated with an elevated risk of NAION. However, its exact mechanism of action remains unclear. Many possible mechanisms underlying the changes in choroidal thickness have been reported. One possible mechanism of choroidal expansion is a change in the tonicity of the choroid.^[6]^ Another way that the choroid could thicken is through changes in vascular permeability, which can cause proteins to move into the extracellular matrix.^[26]^ Other possible mechanisms include autoregulation of human choroids.^[27]^ However, the role of the choroid in the pathogenesis of NAION remains uncertain, despite some of the shared circulation from the posterior ciliary arteries. More in-depth research is required to clarify the possible mechanisms underlying PCT thickening in patients with NAION.

Our meta-analysis had several limitations. First, a small number of studies were included in the analysis, and the quality of evidence was relatively low. Second, the OCT equipment used in each study was not identical, and the choroidal thickness measurements were inconsistent, which could have increased bias. Some studies did not match the refractive state of healthy controls and patients with NAION; furthermore, some studies did not consider diurnal fluctuations in choroidal thickness. Third, OCTA has an advantage over OCT in assessing peripapillary vessel density and structural outcomes in NAION^[28]^; however, OCTA devices are not widely available in China because of their high cost. Finally, this meta-analysis did not pool PCT in acute NAION because of insufficient research data. Future prospective large-scale cohort studies and intervention trials should be conducted to validate our results.

## 5. Conclusion

Our findings suggest that increased MCT and PCT may be important anatomical and physiological features of the eyes of patients with NAION. However, further large-scale prospective studies are required to verify these results.

## Author contributions

**Conceptualization:** Xuejiao Li, Yalong Dang.

**Data curation:** Xuejiao Li, Haoliang Chen.

**Formal analysis:** Haoliang Chen, Yalong Dang.

**Supervision:** Yalong Dang.

**Writing – original draft:** Xuejiao Li.
